# Fibromyalgia among University Students: A Vulnerable Population

**DOI:** 10.31138/mjr.33.4.407

**Published:** 2022-12-31

**Authors:** Georges El Hasbani, Michael Ibrahem, Mohammad Haidous, Monique Chaaya, Imad W. Uthman

**Affiliations:** 1 Department of Internal Medicine, St. Vincent’s Medical Center, Bridgeport, CT, United States of America,; 2 Department of Family Medicine, University of Pittsburgh Medical Centre-Shadyside, Pittsburgh, PA, United States of America,; 3 Department of Internal Medicine, St. Vincent Charity Medical Centre, Cleveland, OH, United States of America,; 4 Department of Epidemiology and Population Health, Faculty of Health Sciences, American University of Beirut, Beirut, Lebanon,; 5 Department of Internal Medicine, American University of Beirut Medical Center, Beirut, Lebanon

**Keywords:** fibromyalgia, university students, prevalence, associations

## Abstract

**Background:**

Fibromyalgia (FM), a complex musculoskeletal disorder, can affect individuals from different genders having different genetic and psychosocial backgrounds. The prevalence of FM depends specifically on the age, gender, and level of stress of the individual. Since the university student body tackles high levels of academic and non-academic stress, we aimed to assess the prevalence and characteristics of FM among such a vulnerable population.

**Methods:**

A survey was sent to participants from two major English-speaking private universities in Lebanon; the American University of Beirut (AUB) and the Lebanese American University (LAU). The survey included the modified American College of Rheumatology (ACR) 2016 criteria, the widespread pain index (WPI), the symptoms severity score (SSS), and the duration of presence of such FM symptoms. In addition, the survey evaluated the presence of other specific musculoskeletal disorders among participants. Nevertheless, a 12-item general healthy questionnaire (GHQ-12) was used to assess the presence of anxiety, depression, social dysfunction, and loss of confidence among participants.

**Results:**

The survey was sent to a total of 2178 students with 184 complete responses (8.45% response rate). The prevalence of FM among the respondents was 13.6%. Students with FM had a significant personal history of a musculoskeletal disorder other than FM and a significant family history of musculoskeletal disorders. The mean SSS score of the target population, including those with FM and those without FM, was 4.5. Patients with FM were significantly in distress and highly symptomatic as measured by GHQ-12 (Unadjusted OR 3.23 [95% CI 1.32–7.95]).

**Conclusion:**

Fibromyalgia seems to be prevalent among university students; in particular, those with other musculoskeletal disorders, those with a family history of musculoskeletal disorders, and those with severe depression and anxiety.

## INTRODUCTION

Fibromyalgia (FM) is a common idiopathic musculoskeletal disorder characterized by widespread pain and multiple cognitive and somatic symptoms.^1^ Several diagnostic criteria exist to aid clinicians in identifying patient with FM.^2,3^ Regardless of the criteria used, widespread or multi-site pain lasting longer than 3 months is the core feature of FM.

The prevalence of FM differs depending on the selected population, the applied diagnostic criteria, and the prevalence of risk factors. For example, whenever the 1990, 2010, and modified 2010 criteria were applied, the average worldwide prevalence obtained was 1.7% (95% confidence interval [95% CI] 0.7–2.8), 1.2% (95% CI 0.3–2.1), and 5.4% (95% CI 4.7–6.1), respectively.^4^ Such prevalence can differ according to the country where the study is performed.^5^ In addition, gender and age play a significant role.^6,7^ Many other significant risk factors exist. For example, body mass index, distress level, smoking status, and residence location were associated with FM assessed among a Lebanese population.^8^

Factors in a certain community, such as physical or mental stress, can also alter the prevalence of FM and worsen its manifestations.^9^ Approximately half of the university student body experiences significant levels of stress in the form of anxiety and/or depression.^10^ These factors make university students vulnerable to an increased risk of developing FM. Although it is reasonable to label the university students as a vulnerable population, this population has been uncommonly studied. Therefore, the aim of our cross-sectional study is to assess the prevalence of FM among university students in Beirut, Lebanon along with the factors that might affect such prevalence.

## METHODS

### Study population and sampling design

This is a cross-sectional study that was conducted between January–June 2020, and which included participants from two major English-speaking private universities in Lebanon; the American University of Beirut (AUB) and the Lebanese American University (LAU). Full-time registered students, between 18 and 25 years of age, and belonging to any major or faculty were eligible to participate in the study.

The list of AUB students from all Faculties was used as a sampling frame. The sample size selected from each faculty was proportionate to its distribution in the university. The sample at LAU was obtained through snowballing. After securing AUB institutional review board (IRB) approval and contacting IRB offices at LAU and getting approval for snowballing, the questionnaire was shared to both universities students who fit the inclusion criteria. The survey was sent to a total of 2178 students through an invitation script sent by LimeSurvey (a free and open source on-line statistical survey web application) to AUB students and by the means of social media platforms (Student groups on Facebook and WhatsApp groups) to LAU students.

The informed consent was displayed in the first page of the electronic survey and included a comprehensive explanation of the research aims, benefits, and inherent risks, as well as the study procedure. The information collected was totally anonymous as no personal data were collected.

### Survey instrument

The survey was constructed based on the modified American College of Rheumatology (ACR) 2016 criteria.^3^ It included the widespread pain index (WPI), a score from 0 to 19 that assesses presence of pain in 5 different regions of the body. The survey also included the symptoms severity score (SSS).^11^ a score from 0 to 12 that assesses whether the person has had any symptoms that interfered with their daily life in the past week, and the duration of presence of such symptoms. In addition, the survey evaluated the presence of other specific musculoskeletal disorders such as back pain or Rheumatoid Arthritis. A general health questionnaire (GHQ-12) was used to assess the presence of anxiety, depression, social dysfunction, and loss of confidence.^12^ Several socio-demographic variables were also evaluated.

## RESULTS

Out of 2178, 184 complete responses were obtained, considering 75% completed survey questions as complete responses. The number of incomplete responses was 102, considering 50% to 75% of completed surveys as incomplete. There were 1892 of no or less than 50 % response.

The prevalence of FM among the population was 25 (13.6%) (**Table 1**). A history of musculoskeletal (MSK) disorder other than FM was more significantly prevalent among students diagnosed with FM. Likewise, a family history of MSK disorder was more significant prevalent among students diagnosed with FM.

**Table 1.** Patient demographics and comparison of history of musculoskeletal disorders between patients with and without fibromyalgia.

**Variable**	**Total**	**Fibromyalgia**		**P value**
	**N (%)**	**Yes** 25 (13.6)	**No** 159 (86.4)	
**Age (Mean, SD)**	20.82 (1.94)	21.13 (2.2)	20.8 (1.9)	0.475
**Gender**				0.103
Males	57(31)	4 (7.0)	53 (93.0)	
Females	123 (65)	20 (16.3)	103 (83.7)	
**BMI (Mean, SD)**	23.28 (4.02)	24.31 (1.05)	23.12 (0.3)	0.289
**BMI**				0.101
< 25	115 (69.7)	16 (12.2)	115 (87.8)	
25 to 29.9	38 (23)	4 (10.5)	34 (89.5)	
30 or more	12 (7.27)	4 (10.5)	8 (89.5)	
**Smoker**				0.356
Yes	32	6 (18.8)	26 (81.2)	
No	151	19 (12.6)	132 (87.4)	
**Income (LBP)**				0.863
< 1,000,000	35 (19.13)	5 (14.3)	30 (85.7)	
1,000,000–1,999,999	37 (20.3)	6 (16.2)	31 (83.8)	
2,000,000 or more	110 (60.44)	14 (12.4)	96 (87.3)	
**Hx of other musculoskeletal disorder (yes)**				0.013*
Yes	39 (21.2)	10 (25.6)	29 (74.4)	
No	145 (78.8)	15 (10.3)	130 (89.7)	
**FHx of other musculoskeletal disorder (yes)**				0.005*
Yes	98 (54.14)	20 (20.4)	78 (79.6)	
No	83 (45.86)	5 (6.0)	78 (94.0)	
**FHx of Fibromyalgia (yes)**				0.077
Yes	18 (9.8)	5 (27.8)	13 (72.2)	
No	165 (90.2)	20 (12.1)	145 (87.9)	

Out of all participants, 59.2% had at least one symptom of the Widespread pain index (WPI) or the Symptoms Severity Score (SSS) (**Table 2**). The mean SSS score of the target population, including those with FM and those without FM, was 4.5 (**Table 3**).

**Table 2.** The distribution of participants according to the widespread pain index (WPI). (Not all N add to 184 because of missing data).

	**N (%) out of 184**
**Have the WPI and SSS symptoms for more than 3 months**	109 (59.2)
**Widespread pain index (WPI)**	
**Left upper region**	
○ L jaw (yes)	19 (10.3)
○ L shoulder girdle (yes)	65 (35.3)
○ L upper arm (yes)	22 (12)
○ L lower arm (yes)	10 (5.4)
**Left lower region**	
○ L hip (buttock/trochanter) (yes)	25 (13.6)
○ L upper leg (yes)	22 (12)
○ L lower leg (yes)	24 (13)
**Right upper region**	
○ R jaw (yes)	20 (10.9)
○ R shoulder girdle (yes)	70 (38.0)
○ R upper arm (yes)	17 (9.2)
○ R lower arm (yes)	12 (6.5)
**Right lower region**	
○ R hip (buttock/trochanter) (yes)	28 (15.2)
○ R upper leg (yes)	20 (10.9)
○ R lower leg (yes)	22 (12.0)
**Axial region**	
○ Neck (yes)	103 (56.0)
○ Upper back (yes)	72 (39.1)
○ Lower back (yes)	94 (51.1)
○ Chest (yes)	18 (9.8)
○ Abdomen (yes)	15 (8.2)
**WPI score (Mean, SD)**	(3.68, 3.42)
**WPI Categories**	
3 or less	105 (57.1)
4–8	63 (32.2)
9–14	14 (7.6)
15 or more	2 (1.1)

**Table 3.** Symptoms Severity Score (SSS) assessment for the target population.

**N (%)**	**0= no problem**	**1= slight or mild problem, often mild or intermittent**	**2= moderate, considerable problem, often present**	**3= severe, pervasive, continuous-life disturbing**
**Fatigue**	40 (21.7)	76 (41.3)	54 (29.3)	14 (7.6)
**Waking unrefreshed**	38 (20.7)	61 (33.2)	64 (34.8)	21 (11.4)
**Cognitive symptoms**	124 (67.4)	32 (17.4)	19 (10.3)	9 (4.9)
				**N (%)**
**In the past week, have you been bothered by any of the following?**				
Headaches (yes)				113 (61.4)
Pain or craps in lower abdomen (yes)				64 (34.8)
Depression (yes)				92 (50.0)
**SSS score (Mean, SD)**				4.5 (2.8)
**SSS Categories**				
4 or less				90 (48.9)
5–8				75 (40.8)
9 or more				19 (10.3)

Anxiety, depression, social dysfunction, and loss of confidence were assessed using the 12-item General Health Questionnaire (GHQ-12).^13^ Almost one third of subjects with FM (32%) had a GHQ score of less than 20 (Not severe, not distressed), 68% had a score of more than 21 (Severe, distressed) (Unadjusted odds ratio of distress 3.23 [1.32–7.95, 95% CI]) (p-value 0.008) (**Table 4**).

**Table 4.** Bivariate association of fibromyalgia and distress.

Fibromyalgia	GHQ 12 (36 points)		Unadjusted OR (95% CI)	P Value
	(20 or more Distressed)	(0–19) Non distressed		0.008^*^
Yes	17 (68)	8 (32)	3.23 (1.32–7.95)	
No	63 (39.7)	96 (60.3)	1.0	

## DISCUSSION

Fibromyalgia (FM) has been assessed among university students, in particular medical students, with a prevalence being less than 12%.^14–16^ The prevalence in our population being 13.6% is higher than that reported in the literature in general, and data from Lebanon in particular.^17^ However, our population included university students from different majors. Stress level might contribute to the difference in FM prevalence, although the stress levels are expected to be higher among medical students than the whole university student body.^18^

The majority of our FM population included females. This finding matches the general population findings.^19^ Furthermore, whether based on the 1990 ACR or the 2010 ACR criteria, gender differences are present among the FM population.^19^ Physical and psychological aetiologies have been suggested, but more studies need to be explored.^20^

Those with FM were more likely to have other associated musculoskeletal disorders. Although the mechanism is unclear yet, the clinical experience and the literature data support the finding that FM is frequently associated with other chronic autoimmune conditions. Since chronic diseases are associated with anxiety and depression, the concomitant presence of FM is expectable.^21^ Besides, the high level of proinflammatory cytokine profile, IL-6, IL-8 and TNF-a as examples, observed in rheumatic conditions could explain some symptoms of FM^21^

Similarly, the presence of FM was significantly associated with family history of musculoskeletal disorders. This might refer to the genetic background of the disease. Genes involved in immunological pathways connected to interleukin-17 and to Type I interferon signatures suggest that autoimmunity plays a role in the disease.^22^

General Health Questionnaire (GHQ) has been used in FM patients to explore psychiatric comorbidity. 23 Psychiatric conditions, depression and anxiety in particular, might overlap with FM.^24,25^ Similar to what is expected from literature data, a majority of our cohort’s FM population had highly symptomatic classification according to the GHQ-12; scoring more than or equal to 20.^26^ Besides anxiety and depression, there is also evidence of high prevalence of neuroticism, perfectionism, stress, and anger among FM patients.^27^

The main limitation of our study was its design being a cross-sectional study. In addition, the response rate was low. There might be also some potential for reporting bias, depending on the students’ perception of the questions. Moreover, people having musculoskeletal complaints are more likely to be interested in filling in the questionnaire which could partly explain the higher prevalence of FM among our university population in Lebanon compared to other studies. Despite these limitations, our study adds significant importance to the literature as it is the first to report the prevalence of FM among university students as a whole body regardless of the majors. We anticipate future studies that can stratify the university student population into different schools and majors to compare FM prevalence and complications.

## CONCLUSION

The study revealed that Fibromyalgia is prevalent university students in Lebanon and its magnitude higher than in other students population. Similar to the general population, the disease is more prevalent among females than males. The presence of FM seems to be associated with the presence of other musculoskeletal disorders as well as a family history of musculoskeletal disorders. This gives an insight towards the genetic and immunological background of FM. Clinicians in general, and rheumatologists in particular, should be aware that FM is a potential aetiology for a university student presenting with aches and pains. FM seems also to be associated with a higher threshold of psychiatric illnesses, although more studies are needed to assess the predisposition of chronic pain to psychiatric illness and vice versa.
